# Reanalysis and optimisation of bioinformatic pipelines is critical for mutation detection

**DOI:** 10.1002/humu.23699

**Published:** 2019-01-31

**Authors:** Mark J Cowley, Yu‐Chi Liu, Karen L. Oliver, Gemma Carvill, Candace T. Myers, Velimir Gayevskiy, Martin Delatycki, Danique R.M. Vlaskamp, Ying Zhu, Heather Mefford, Michael F. Buckley, Melanie Bahlo, Ingrid E. Scheffer, Marcel E. Dinger, Tony Roscioli

**Affiliations:** ^1^ Kinghorn Centre for Clinical Genomics Garvan Institute of Medical Research Darlinghurst NSW Australia; ^2^ St Vincent's Clinical School University of New South Wales Darlinghurst Australia; ^3^ Population Health and Immunity Division Walter and Eliza Hall Institute Melbourne Australia; ^4^ Epilepsy Research Centre, Department of Medicine University of Melbourne, Austin Health Heidelberg Australia; ^5^ Department of Medical Biology The University of Melbourne Parkville Victoria Australia; ^6^ Ken and Ruth Davee Department of Neurology Northwestern University Feinberg School of Medicine Chicago IL; ^7^ Department of Pediatrics University of Washington Seattle WA; ^8^ Austin Health Melbourne Australia; ^9^ Department of Medical Genetics Royal North Shore Hospital St Leonards Australia; ^10^ NSW Health Pathology Randwick Sydney Australia; ^11^ Florey Institute Melbourne Australia; ^12^ Department of Paediatrics University of Melbourne Royal Children's Hospital Parkville Australia; ^13^ Centre for Clinical Genetics Sydney Children's Hospital Randwick Australia; ^14^ Prince of Wales Clinical School University of New South Wales Sydney Australia; ^15^ Neuroscience Research Australia University of New South Wales Randwick Sydney Australia

**Keywords:** clinical bioinformatics, de novo, developmental and epileptic encephalopathy, whole genome sequencing

## Abstract

Rapid advances in genomic technologies have facilitated the identification pathogenic variants causing human disease. We report siblings with developmental and epileptic encephalopathy due to a novel, shared heterozygous pathogenic 13 bp duplication in *SYNGAP1* (c.435_447dup, p.(L150Vfs*6)) that was identified by whole genome sequencing (WGS). The pathogenic variant had escaped earlier detection via two methodologies: whole exome sequencing and high‐depth targeted sequencing. Both technologies had produced reads carrying the variant, however, they were either not aligned due to the size of the insertion or aligned to multiple major histocompatibility complex (MHC) regions in the hg19 reference genome, making the critical reads unavailable for variant calling. The WGS pipeline followed different protocols, including alignment of reads to the GRCh37 reference genome, which lacks the additional MHC contigs. Our findings highlight the benefit of using orthogonal clinical bioinformatic pipelines and all relevant inheritance patterns to re‐analyze genomic data in undiagnosed patients.

Two sisters with developmental and epileptic encephalopathy (Scheffer et al., 2017) were referred for genomic testing. The 7‐year‐old proband was the first child to non‐consanguineous parents, an English father and Australian mother, both of Ashkenazi Jewish origin. There was no family history of epilepsy or intellectual disability. Following an unremarkable perinatal history, her vocalization regressed at age 6 months and her subsequent development was delayed, walking at 22 months and speaking single words at 2.5 years. At age 7 years, she had a total of 100 single words, but lacked word combinations. She was assessed as having an intellectual disability, behavioral problems with tantrums, and trichotillomania. She also had some features consistent with autism spectrum disorder and obsessive behaviors.

Absence seizures with eyelid myoclonus began at 8 months. She then developed drop attacks secondary to myoclonic‐atonic seizures. The seizures remained refractory to treatment with up to 50 absence seizures and eyelid myoclonus occurring per hour despite multiple antiepileptic medications. EEG studies showed frequent 3 Hz generalized spike‐wave activity and prominent photosensitivity.

Her sister was 2 years younger and presented with seizures at age 12 months; she followed a similar clinical, but milder, course (Table 1). Her motor milestones were normal, but speech acquisition was delayed with single words at 22 months. At 5 years, she could combine words.

**Table 1:** Phenotypic features present in the proband and affected sibling

Clinical features	Proband	Sibling
Absence seizures with eyelid myoclonia (HP:0011149)	Yes	Yes
Myoclonic atonic seizures (HP:0011170)	Yes	Yes
Myoclonic seizures (HP:0002123)	Yes	Yes
EEG with generalized epileptiform discharges (HP:0011198)	Yes	Yes
EEG with photoparoxysmal response (HP:0010852)	Yes	Yes
Delayed developmental milestones (HP:0001263)	Yes	Yes
Developmental regression (HP:0002376)	Yes	No
Language impairment (HP:0002463)	Yes	Yes
Intellectual disability, moderate (HP:0002342)	Yes	Yes
Novel clinical features		
Autism spectrum disorder (HP:0000729)	No	Yes
Trichotillomania (HP:0012167)	Yes	No
Severe temper tantrums (HP:0025162)	Yes	No
Echolalia (HP:0010529)	No	Yes
Trouble sleeping (HP:0002360)	Yes	Yes
Pica (HP:011856)	Yes	Yes
Hypotonia (HP:0001290)	Yes	Yes
Ataxic gait (HP:0002066)	Yes	Yes
Hearing loss (HP:0000365)	Yes^a^	Yes^a^

These features were absent from both parents. Human Phenotype Ontology (HPO) terms are listed. EEG, electroencephalography.

a Unrelated to their inherited genetic condition.

Both girls had hearing loss due to chronic ear infections. Their vision was normal. Overall, the sisters had an epilepsy phenotype that had features of two well‐established epilepsy syndromes: epilepsy with myoclonic‐atonic seizures and epilepsy with eyelid myoclonus.

An extensive diagnostic workup did not identify an etiology. A SNP microarray detected a de novo 700 Kb duplication at chrXq27.1, including *SOX3*, which was not thought to be contributory, as well as two small regions of homozygosity on chromosomes 1 and 9. Mitochondrial sequencing for 22 mtDNA and three common *POLG* variants (Uusimaa et al., 2013) was normal. Methylation studies for Angelman Syndrome were normal. Biochemical studies including serum lactate, pyruvate, and CSF amino acids, glucose, neurotransmitters, and methyltetrahydrofolate were normal. Lysosomal enzymes, carnitine studies, serum vitamin D levels, selenium, red cell folate, active B12, transferrin isoforms, and iron levels were normal. A brain MRI performed on the proband was normal.

High throughput targeted sequencing of 65 epilepsy genes using molecular inversion probes (MIPs; Carvill et al., 2013), was performed on DNA from the family quartet (both girls and their parents) in 2014. Note that 100 bp paired end reads were aligned to a custom hg19 reference genome containing only chromosomes 1–22, X, Y, chrM, using bwa sampe (v0.5.9‐r16; Li & Durbin, 2009), standard settings, and variant analysis and filtration as described (Carvill et al., 2013). No pathogenic variants were identified.

Whole exome sequencing (WES) was performed to ∼50× depth on the quartet at the Australian Genome Research Facility in 2015. Reads were aligned to hg19 using bwa mem (v0.7.10; Li, 2013), with variant analysis and filtration as described (Y.‐C. Liu et al., 2016). After in silico filtering for genes matching the patient phenotype, as well as for variants segregating in the affected individuals, no plausible candidate genes were identified.

WGS was performed to 28–40× depth using Illumina HiSeq X at the Kinghorn Centre for Clinical Genomics, Australia, on the quartet, in 2016. Genomic data were processed according to the GATK best practices guidelines (Van der Auwera et al., 2013), using GATK (v3.3; (DePristo et al., 2011; McKenna et al., 2010), as previously described (Mallawaarachchi et al., 2016). Reads were aligned to the b37d5 (1000 genomes + decoy) reference genome using bwa mem (v0.7.10; (Li, 2013)) followed by indel realignment and base quality recalibration. Single nucleotide variants and short insertions and deletions were joint‐called using HaplotypeCaller in gVCF mode, with variant quality score recalibration. Variants were annotated using VEP (v79; McLaren et al., 2016), dbNSFP (v2.0; X. Liu, Jian, & Boerwinkle, 2013) and CADD (v1.0; Kircher et al., 2014), converted to a Gemini database (v0.11.0; Paila, Chapman, Kirchner, & Quinlan, 2013) and filtered using *Seave* (Gayevskiy, Roscioli, Dinger, & Cowley, 2018). The same heterozygous de novo frameshift variant in exon 5 of *SYNGAP1* was identified in both siblings using a shared de novo pattern of inheritance, which was consistent with gonadal mosaicism in one parent (Supporting Information Table S1). There were no additional candidate variants in relevant genes that segregated with the disease for an autosomal recessive, autosomal dominant or shared de novo model.

A heterozygous 13 bp duplication in *SYNGAP1* (NM_006772.2:c.435_447dup, NP_006763.2:p.(Leu150LysfsTer6), Supporting Information Table S1) was identified by WGS in both girls. This variant was absent in DNA extracted from the peripheral blood from both parents, consistent with gonadal mosaicism as the most likely explanation. The variant was absent from ExAC (Lek et al., 2015), GnomAD, and 1000 genomes (1000 Genomes Project Consortium et al., 2015). While this variant has not been reported in the literature, ClinVar or OMIM, it is within the N‐terminal Pleckstrin homology domain, close to many previously reported pathogenic loss of function variants (Figure 1a). *SYNGAP1* has a pLI score (Samocha et al., 2014) of 1.0, indicating it is intolerant of loss of function variation. The variant was validated by Sanger sequencing in a clinically accredited laboratory and reported as an ACMG class V pathogenic variant (Supporting Information Table S1).

**Figure 1:**
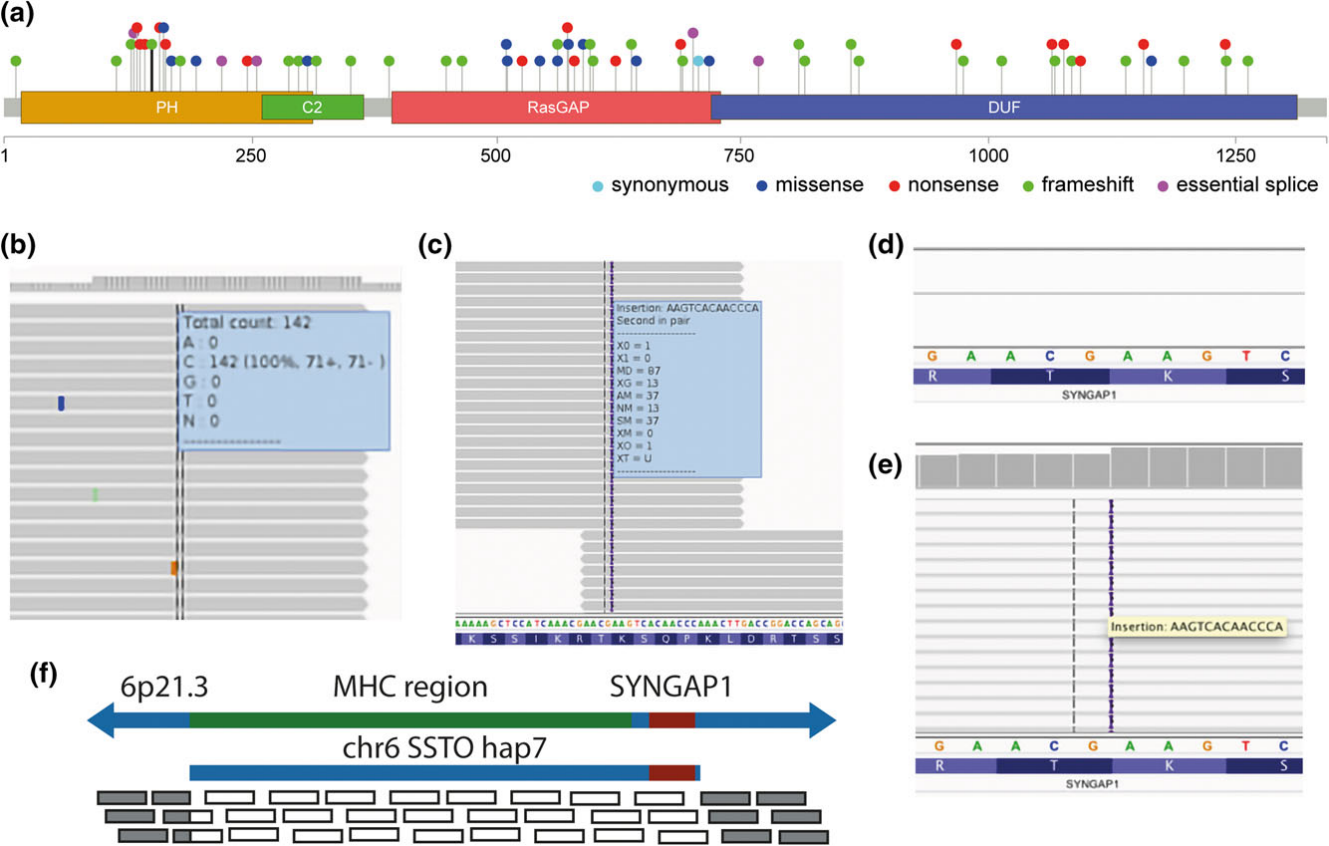
(a) Using whole genome sequencing (WGS), we identified a pathogenic SYNGAP1 p.L150Vfs*6 variant (black stalk within PH domain). All 65 pathogenic or likely pathogenic SYNGAP1 variants reported in ClinVar (accessed 1st April, 2018) are colored by mutation type. Figure created using lollipops, with domains from InterPro. PH: pleckstrin homology domain; C2: C2 domain; RasGAP: rho GTPase activation protein domain; DUF: domain of unknown function (DUF3498). (b) Standard analysis of targeted molecular inversion probes (MIPs) sequencing of epilepsy genes including SYNGAP1 identified 142 reads covering the same region, none of which carried the mutation. (c) After extending the maximum number of gap extensions (–e 20) during alignment, reads carrying the 13 bp duplication aligned correctly to SYNGAP1 exon 5. (d) Whole exome sequencing (WES) revealed zero reads covering SYNGAP1 with mapping quality >20. (e) Removing the mapping quality filter revealed an average of 91 reads, including 50 reads carrying the 13 bp duplication. (f) SYNGAP1 is distal to the major histocompatibility complex (MHC) on chr6p21.3. One of the MHC contigs included in the hg19 reference genome, chr6_ssto_hap7 includes SYNGAP1. The mapping quality score for reads that align to both chr6, and chr6_ssto_hap7 were penalized to zero (translucent reads) whereas reads that map only to chr6 have high mapping quality (grey reads)

Synaptic GTPase–activating protein 1 (*SYNGAP1)* encodes a brain‐specific synaptic Ras GTPase activating protein that suppresses signaling pathways linked to NMDA receptor (NMDAR)‐mediated synaptic plasticity and AMPA receptor (AMPAR) membrane insertion (McKusick, 2007). De novo truncating variants in *SYNGAP1* leading to haploinsufficiency were first identified in individuals with moderate‐to‐severe intellectual disability (Hamdan et al., 2009), and we identified it as a cause of developmental and epileptic encephalopathy (Carvill et al., 2013). De novo mutations in *SYNGAP1* are a relatively frequent cause of developmental delay (Deciphering Developmental Disorders Study et al., 2017). Forty of the 64 pathogenic or likely pathogenic *SYNGAP1* variants currently reported in ClinVar are associated with the OMIM disorder Mental Retardation, Autosomal Dominant 5 (MIM 612621).

WGS differed from previous genetic testing in several ways, including no potential for capture or design bias, longer read length (2 × 150 bp), the reference genome, and versions of alignment and variant calling software. After confirming that both the targeted MIPs panel and the WES targeted this region of *SYNGAP1*, we investigated which factors led to the variant being missed by previous testing.

In the MIPs targeted sequencing data, good gene coverage of *SYNGAP1* exon 5 was observed, with a read depth of >142× in the older sister (Figure 1b). Due to the use of an older read aligner (i.e., bwa sampe v0.5.9‐r16), and the high ratio of 13 bp mutation to 100 bp read length, we hypothesized that the reads supporting the duplication may have been misaligned. The reads were re‐aligned to the reference genome, increasing the maximum number of gap extensions (–e 20), which resulted in the reads that carried the variant being appropriately aligned from two overlapping amplicons (Figure 1c). Updating the aligner to bwa mem v0.7.15 also correctly aligned the reads carrying the mutation using default settings (not shown).

In the WES dataset, we observed no reads across *SYNGAP1* with mapping quality >20 (Figure 1d). Removing the mapping quality filter revealed an average read depth of 91× across exon 5 (Figure 1e), consistent with read alignment to multiple regions of the reference genome. Importantly this also identified that there were reads carrying the heterozygous pathogenic variant.

The hg19 version of the reference genome from UCSC contains seven additional sequences at the 6p13 locus, to capture the extensive genetic variation in the major histocompatibility complex (MHC) (Lam, Tay, Wang, Xiao, & Ren, 2015). *SYNGAP1* is found centromeric to one of the common HLA haplotypes, A1‐B8‐DR3‐DQ2 (Horton et al., 2008), represented by the chr6_ssto_hap7 contig (Figure 1F). Consequently, the sequencing reads from *SYNGAP1* mapped perfectly to both chr6p21.3 and chr6_ssto_hap7, and their mapping quality scores were set to zero (Figure 1f), making these reads invisible to variant identification tools. The pathogenic variant was identified using default detection settings once the reads were re‐aligned to the GRCh37 reference genome that lacks the additional MHC contigs, or to GRCh38 with an ‘alt‐aware’ read aligner, bwa mem (v0.7.12), which assigned the correct mapping quality scores to the reads.

In summary, we identified a shared heterozygous *SYNGAP1* p.(L150Vfs*6) variant (ACMG class V, pathogenic) in two siblings with developmental and epileptic encephalopathy through an improved WGS bioinformatics pipeline, and consideration of multiple modes of inheritance. Despite having greater than 40 high quality reads supporting this variant, it was missed by both high‐depth targeted MIPs sequencing and WES, due to two different technical reasons, principally based on read alignment issues. Even as many groups converge upon similar BWA‐GATK best practice pipelines (Van der Auwera et al., 2013), there are still many variables including choice of reference genome, base and variant quality recalibration settings, variant annotation and filtration tools, and versions of software that influence variant detection. The differences between the reference genome versions have been recently summarized (Li, 2017).

This case report focused only on *SYNGAP1*, therefore, we investigated which additional genes may be affected by the same mapping issues. There are 245 genes represented by at least one overlapping MHC contig, including 31 genes associated with human diseases (Supporting Information Table S2). Among these, *COL11A2* is associated with Autosomal Dominant Stickler Syndrome (MIM 604841), Marshall Syndrome (MIM 154780), and Autosomal recessive fibrochondrogenesis (MIM 228520). Neuraminidase 1 (*NEU1*) is associated with autosomal recessive sialidosis (MIM 256550), a lysosomal storage disease affecting the nervous system. These, and others reviewed in Supporting Information Table S2 are similarly affected by the read mapping issues reported here and so should have appropriate coverage analysis performed. We note that the duplication of sequences in the MHC region, or other ‘alt’ contigs are distinct from other truly duplicated regions of the genome, including highly homologous pseudogenes, or the pseudoautosomal regions. Innovative approaches are beginning to resolve some regions of the genome previously classified as inaccessible to short‐read sequencing, e.g., *SMN1* and *SMN2* (Feng et al., 2017).

Updating analysis pipelines has been shown to increase the diagnostic yield on systematic retrospective re‐analyses (Wright et al., 2018). In a recent multi‐laboratory study of challenging variants, bioinformatic errors were a major cause of considerable inter‐laboratory discordance, even among clinical laboratories (Lincoln et al., ). Our results suggest that updating the reference genome and aligner versions should be considered in any retrospective re‐analyses of undiagnosed patient genome data. Additionally, alignment‐free (Ostrander et al., 2018), or deep‐learning based variant calling methods (Poplin et al., 2018) may be considered as maximally orthogonal approaches for re‐analyzing data. Initiatives such as the Broad Institute's “Functional Equivalence” specification. PrecisionFDA (Petrone, 2016), Genome in a Bottle (Zook, Catoe et al., 2016; Zook, Chapman et al., 2014) and the DREAM challenges (Boutros et al., 2014; Zook, Catoe et al., 2016; Zook, Chapman et al., 2014) provide objective feedback as to the performance of bioinformatic pipelines and help labs know if their pipelines may be underperforming, and warrant updating.

This case report suggests that lack of diagnosis with different genomic technologies may occur due to technical limitations and that clinical genomic re‐analysis including all potential inheritance patterns and the use of orthogonal and updated bioinformatic pipelines may identify previously undetected pathogenic variants. Comprehensive assessment of read coverage across all disease‐relevant genes should be performed in parallel with variant detection pipelines to highlight poorly covered genes with potential pathogenic variation.

## Supporting information

Supp. Table S1. Summary of genomic findings
Supp. Table S2. Additional genes in the MHC region linked to disease.
