# Supplementary material for: Reanalysis and optimisation of bioinformatic pipelines is critical for mutation detection
Source: Hum Mutat. 2019 Jan 31;40(4):374–9. doi: 10.1002/humu.23699 (PMC6492103; doi:10.1002/humu.23699)
Supplement: Supplementary file 1 — Supp. Table S1. Summary of genomic findings Supp. Table S2. Additional genes in the MHC region linked to disease. [file HUMU-40-374-s001.pdf]

Supp. Table S1. Summary of genomic findings

**Gene**	*SYNGAP1*
**Genomic location**	chr6:33400507-33400519 (GRCh37)<br>chr6:33432730-33432742 (GRCh38)
**HGVS cDNA**	NM_006772.2:c.435_447dup
**HGVS protein**	NP_006763.2:p.(Leu150LysfsTer6)
**Inheritance and Zygosity**	*de novo* heterozygous
**Variant interpretation**	ACMG class V Pathogenic

Supp. Table S2. Additional genes in the MHC region linked to disease.
Thirty-one genes which overlapped any of the MHC contigs represented in the hg19 reference genome, with links to at least one disease. Genes were annotated by OMIM terms (denoted by MIM numbers), then complemented by annotations from OrphaNet (denoted by ORPHA numbers), and ClinVar cards (denoted by 'ClinVar Cards', but no accession).

**Gene**	**Disorders**
C2	C2 deficiency, (MIM: 217000)<br>Macular degeneration, age-related, 14, reduced risk of, (MIM: 615489)
C4B	C4B deficiency, (MIM: 614379)
C6orf25	Thrombocytopenia, anemia, and myelofibrosis, (MIM: 617441)
CDSN	Hypotrichosis 2, (MIM: 146520)<br>Peeling skin syndrome 1, (MIM: 270300)
CFB	Macular degeneration, age-related, 14, reduced risk of, (MIM: 615489)<br>Hemolytic uremic syndrome, atypical, susceptibility to, 4, (MIM: 612924)<br>Complement factor B deficiency, (MIM: 615561)
COL11A2	Otospondylomegaepiphyseal dysplasia, autosomal recessive, (MIM: 215150)<br>Deafness, autosomal dominant 13, (MIM: 601868)<br>Deafness, autosomal recessive 53, (MIM: 609706)<br>Fibrochondrogenesis 2, (MIM: 614524)<br>Marshall Syndrome, (MIM: 154780)<br>Otospondylomegaepiphyseal dysplasia, autosomal dominant, (MIM: 184840, ORPHA: 3450)<br>Stickler Syndrome, type 2 (MIM, 604841)

CYP21A2	Adrenal hyperplasia, congenital, due to 21-hydroxylase deficiency, (MIM: 201910)<br>Hyperandrogenism, nonclassic type, due to 21-hydroxylase deficiency, (MIM: 201910)
CSNK2B	Intellectual disability and seizures (ClinVar Cards)
DAXX	Metastatic pancreatic neuroendocrine tumours (ClinVar Cards)
HLA-A	Hypersensitivity syndrome, carbamazepine-induced, susceptibility to, (MIM: 608579)
HLA-DPB1	Major susceptibility factor in Chronic beryllium disease (ORPHA: 133)<br>Major susceptibility factor in Granulomatosis with polyangiitis (ORPHA: 900)
LTA	Myocardial infarction, susceptibility to, (MIM: 608446)<br>Psoriatic arthritis, susceptibility to, (MIM: 607507)<br>Leprosy, susceptibility to, 4, (MIM: 610988)
MOG	Narcolepsy 7, (MIM: 614250)
MSH5	Premature ovarian failure 13, (MIM: 617442)
NEU1	Sialidosis, type I, (MIM: 256550)<br>Sialidosis, type II, (MIM: 256550)
OR2J3	C3HEX, ability to smell, (MIM: 615082)
PSMB8	Proteasome-associated autoinflammatory syndrome 1 and digenic forms, (MIM: 256040)<br>Nakajo-Nishimura syndrome, autosomal recessive (ORPHA: 2615)<br>CANDLE syndrome, autosomal recessive (ORPHA: 325004)
PSMB9	Proteasome-associated autoinflammatory syndrome 3, digenic, (MIM: 617591)
SKIV2L	Trichohepatoenteric syndrome 2, (MIM: 614602)<br>Syndromic diarrhea, autosomal recessive (ORPHA: 84064)
SLC44A4	Deafness, autosomal dominant 72, (MIM: 617606)
STK19	Squamous cell carcinoma of the skin (ClinVar Cards)<br>Malignant melanoma of skin (ClinVar Cards)
SYNGAP1	Mental retardation, autosomal dominant 5, (MIM: 612621)<br>Global developmental delay (ClinVar Cards)<br>Cerebellar ataxia (ClinVar Cards)<br>Epileptic encephalopathy (ClinVar Cards)
TAP1	Bare lymphocyte syndrome, type I, (MIM: 604571)<br>Immunodeficiency by defective expression of HLA class 1, Autosomal Recessive (ORPHA: 34592)
TAP2	Immunodeficiency by defective expression of HLA class 1, Autosomal Recessive (ORPHA: 34592)

TAPBP	Bare lymphocyte syndrome, type I, (MIM: 604571)<br>Immunodeficiency by defective expression of HLA class 1, Autosomal Recessive (ORPHA: 34592)
TNF	Malaria, cerebral, susceptibility to, (MIM: 611162)<br>Asthma, susceptibility to, (MIM: 600807)<br>Migraine without aura, susceptibility to, (MIM: 157300)
TNXB	Ehlers-Danlos syndrome, classic-like, 1 (MIM: 606408)<br>Vesicoureteral reflux 8, (MIM: 615963)
TUBB	Cortical dysplasia, complex, with other brain malformations 6, (MIM: 615771)<br>Symmetric circumferential skin creases, congenital, 1, (MIM: 156610)
VARS	Neurodevelopmental disorder with microcephaly, seizures, and cortical atrophy, (MIM: 617802)
VARS2	Combined oxidative phosphorylation deficiency 20, (MIM: 615917)
ZFP57	Diabetes mellitus, transient neonatal, 1, (MIM: 601410)
